# Detection of California Serogroup Orthobunyavirus antibodies and Inkoo virus RNA in patients, Finland

**DOI:** 10.1017/S0950268825100289

**Published:** 2025-07-17

**Authors:** Eveliina Ekström, Katariina Kaansalo, Maija T. Suvanto, Mira Utriainen, Niina Putkuri, Olli Vapalahti, Hannimari Kallio-Kokko, Eili Huhtamo, Anne J. Jääskeläinen

**Affiliations:** 1 HUS Diagnostic Center, Virology and Immunology, Helsinki University Hospital, Helsinki, Finland; 2 Department of Virology, University of Helsinki, Helsinki, Finland; 3 Department of Veterinary Biosciences, University of Helsinki, Helsinki, Finland; 4 Finnish Red Cross, Blood Service, Vantaa, Finland

**Keywords:** Chatanga virus, Inkoo virus, neutralization assay, orthobunyavirus, PCR, serology

## Abstract

Mosquito-borne California serogroup orthobunyaviruses Inkoo (INKV) and Chatanga (CHATV) are known to be endemic in Finland with a high seroprevalence. We developed a novel multiplexed reverse transcription quantitative polymerase chain reaction method for discriminating between the INKV and CHATV. This assay was used along with traditional serological tests to study a set of summertime patients during the years 2021, 2023, and 2024 to assess the epidemiology and prevalence of acute INKV and CHATV infections in Finland. Altogether, 1470 samples were screened, and there were 16 patients who had an acute infection based on serological findings and/or nucleic acid test. The orthobunyavirus-IgG seroprevalences were 18% (2021), 20% (2023), and 30% (2024), being lower than that in studies from 20 years ago. Neutralization tests were carried out, and all but one acute case had more than four-fold higher titre to INVK vs. CHATV, indicating specificity to INKV infection. The results suggest that epidemiology has changed from previous studies, and INKV should be considered a causative agent of summertime infections in Finland. The symptom diversity in mild disease outcomes should be studied to guide orthobunyavirus recognition by clinicians. The use of molecular assay discriminating INKV and CHATV aids in understanding disease associations.

## Introduction

In northern Europe, and more precisely in Fennoscandia, there are currently only a few pathogenic and endemic mosquito-borne viruses: an *Alphavirus*, Sindbis virus (SINV), and two *Orthobunyaviruses*: Inkoo virus (INKV) and Chatanga virus (CHATV; synonym for Khatanga virus) [1]. Both INKV and CHATV belong to the California serogroup (CSG) in genus *Orthobunyavirus*, in the family Peribunyaviridae. Orthobunyaviruses are vector-borne enveloped viruses with a tripartite negative-sense RNA genome. The SINV, INKV, and CHATV are maintained in sylvatic cycles among wild vertebrate hosts and mosquitoes. Humans are considered dead-end hosts, and only SINV is known to cause outbreaks of human disease.

Based on phylogenetic studies, the closest relative to INKV is the Jamestown Canyon virus, which is circulating in the United States [2, 3]. In Finland, the INKV was isolated in 1964 from *Ochlerotatus communis* and *Ochlerotatus punctor* mosquitoes [4] and CHATV in 2007 from pools of *Ochlerotatus* and *O./Aedes* mosquitoes [5]. In Sweden, INKV has also been detected in larvae of *O. communis* [6], suggesting that *O. communis* is the main vector for INKV. While *O. communis* is the most abundant mosquito in Finland, it is not found in all parts of Lapland, whereas *O. punctor* can be found all over Finland, even in Lapland [7].

During the summer and autumn seasons, the most common mosquito-borne viral disease detected in Finland is the Pogosta disease, aka Ockelbo disease in Sweden, and Karelian fever in Russia, caused by SINV [8]. Acute infections with INKV, CHATV, or SINV, in the early phase, can manifest as a non-specific febrile illness. The symptoms of SINV infections are well recognized, and the aetiology is usually known in Finland. However, infections caused by INKV and CHATV, with either mild or severe outcomes, are not suspected or detected by clinicians in Finland. At the moment, their public health impact is unknown. Severe manifestations of INKV and CHATV infections, including those with central nervous system (CNS) symptoms, have been reported in Finland [9]. In short, in the acute phase, the symptoms of INKV, CHATV, or SINV infections are all non-specific, including fever with possible headache, nausea, and fatigue. Later in the SINV infection, they are followed by rash, inflammation, and arthralgia [10, 11], while the INKV and CHATV infections cause influenza-like symptoms, disorientation, and CNS symptoms [9].

In this study, we collected human samples during summer and autumn, reflecting the active mosquito season in Finland during the years 2021, 2023, and 2024. All these samples originated from patients assumed to have mosquito contact as they were all originally suspected of the mosquito-borne Pogosta disease. As a limitation, no demographic or patient data were available for this study. Sera were screened for anti-CSG orthobunyavirus antibodies using an indirect immunofluorescence assay (IFA) to detect exposure to INKV or CHATV. All acute cases detected by IFA were confirmed using INKV and CHATV neutralization assays. Furthermore, from all samples (*N* = 1470), nucleic acids were extracted, and INKV or CHATV viraemia was screened using developed and validated INKV-CHAT multiplex real-time reverse transcription quantitative polymerase chain reaction (MPLEX-RT-qPCR). If positive for viral RNA, virus isolations and sequencing approaches were attempted.

## Materials and methods

### Patient samples

**Figure 1.**
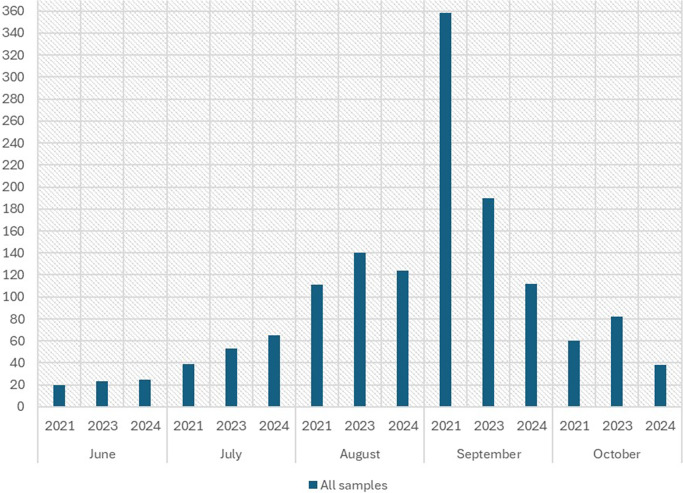
Altogether, 588, 488, and 394 serum samples (*N* = 1470) were collected from the years 2021, 2023, and 2024, respectively. The sample distribution per month (June–October) and year has been presented in this figure.

In total, 588, 488, and 394 serum samples (*N* = 1470) from years 2021, 2023, and 2024, respectively, were studied for orthobunyavirus infections (Figure 1; sample distribution per month). These were collected from June until October and originally sent and tested for anti-SINV antibodies at HUS Diagnostic Center (Helsinki, Finland). All samples were anonymous and included in the study without any selection. Samples were studied anonymously, and no demographic data were available according to research and ethic permits of 159/HUS/151/2022 and 3-HUS/31/2024 (HUS Diagnostic Center). Nucleic acids were extracted from 581, 488, and 394 serum samples (*N* = 1463) from years 2021, 2023, and 2024, respectively, using the MagNA Pure 96 system (Roche) and total nucleic acid extraction kit according to the manufacturer’s instructions, and stored at −70 °C. From all 588 samples collected in 2021, only 581 had enough sample volume available to be extracted using the MagNA Pure 96 system (Roche).

### Indirect IFA and microneutralization test (MNT)

From all 1470 serum samples, anti-CSG orthobunyavirus IgG antibodies were screened using INKV IFA ([12]; HUS Diagnostic Center). As CSG orthobunyaviruses cross-react in serological testing, all samples positive using anti-INKV IgG IFA were further tested for IgM antibodies using anti-INKV IgM IFA (HUS Diagnostic Center). The IFA results were interpreted as indicative of old immunity with diffuse IgG fluorescence pattern and no IgM detected, and acute or recent infection if the IgG and IgM were positive and the fluorescence pattern was granular, typical for bunyaviruses [12, 13, 14]. To reliably discriminate anti-INKV and CHATV antibodies from each other, MNTs against INKV and CHATV were carried out for all samples with acute infection (IgG and IgM positives) detected by IFA, 30 samples with old anti-CSG immunity (IgG positive, but IgM negative), and one negative serum from this study, in addition to cell culture media and anti-CSG antibody-negative serum to control the performance of IFA and MNT.

The MNT was carried out using INKV (strain KN3641, in-house [3]) and CHATV (strain Möhkö M07-1, in-house [5]) on a 96-well plate format. In brief, patient sera were inactivated at 56 °C for 30 min and diluted into cell culture media (minimum essential medium (MEM) with added 2% foetal bovine serum and glutamine, penicillin, and streptomycin). Virus was added to the mixture (multiplicity of infection (MOI 0.5)) and incubated with the serum dilutions at +37 °C for 1 h. The serum–virus mixture was added to confluent Vero E6 cells. After 2 days of incubation at +37 °C, 5% CO_2,_ the wells were fixed with 10% formaldehyde (in water) and stained with crystal violet solution. The controls included a previously known INKV seropositive human sample and a virus control (no serum added). The result was interpreted as positive for neutralization if stained cells were visible in the well. The wells that had no visible cell layer left, as in the virus control well, were considered negative for virus neutralization.

### INKV-CHATV MPLEX-RT-qPCR

MPLEX-RT-qPCR was designed for detection of INKV and CHATV nucleic acids using Primer3web (version 4.1.0) [15] and ClustalW (GenomeNet) and L-segment, which is a highly conserved gene area among different INKV and CHATV strains. For INKV-CHATV MPLEX-RT-qPCR, Invitrogen™ SuperScript™ III One-Step RT-PCR System with Platinum™ Taq DNA Polymerase (Thermo Fisher Scientific, Vantaa, Finland) with final concentration of 600 nM of INKV-FW (5′-TGATTGGCAAACATCAAGGC-3′) and INKV-RV (5′-A(G/A)TTCCCTGGGTATGTTGT(A/G)ACT-3′), 300 nM of INKV-probe1 (6-FAM-MGBNFQ; 5′-C(C/T)AGGCATGGTTTGAAATTTGA-3′) and INKV-probe2 (6-FAM-MGBNFQ; 5′-TCACTAGGAAGACACCAGAAAA-3′), and for CHATV, 400 nM of CHAT-FW1 (5′-ACGCCACGGTCTTAAGTTTG-3′) and CHAT-FW2 (5′-ACGCCATGGTCTTAAATTCG-3′), 500 nM CHAT-RV (5′-TGCCATGTGTTC(T/C)TC(G/A)TTTCT-3′), and 300 nM of CHAT-probe1 (VIC-MGBNFQ; 5′-AGAAGGAAAGACCGGCATCA-3′) and CHAT-probe2 (VIC-MGBNFQ; 5′-AGAAAGAAGGATCGACATCA-3′) were used, with nucleic acid template of 7 μl. The PCR program consisted of reverse transcription at 50 °C for 30 min, followed by 95 °C for 2 min, and 45 cycles at 95 °C for 15 s, at 60 °C for 50 s.

Commercial plasmid positive controls for MPLEX-RT-qPCR were used for INKV strain KN3641 (Finland; NCBI Seq ID: EU789573.1), strain Lovanger (Sweden: NCBI seq ID: KX554937.1) and CHATV strain LEIV-18733 (NCBI Seq ID: HQ734817.1), and strain Möhkö (NCBI Seq ID: KF719235.1) (IDT Technologies, USA). The limit of detection was determined using quantified plasmid controls for INKV (strain KN3641) and CHATV (strain Möhkö) with ten parallel reactions and Probit (IBM SPSS Software), and furthermore, intra-assay reproducibility was determined. The assay specificity was evaluated with 16 non-INKV and CHATV viral RNAs, 20 negative sera, and 20 EDTA-blood samples (Table 2).

### Virus isolation trials

Virus isolations were attempted from two patient samples, which were positive for INKV RNA, using baby hamster kidney (BHK-21, ATCC: CCL-10) and green monkey kidney (Vero E6, ATCC CRL-1586) cells in 25 cm^2^ cell culture flasks. In brief, the cell monolayer was rinsed three times with PBS + Penicillin–Streptomycin (PenStrep, 10000 U/ml of penicillin and 10000 U/ml streptomycin), and the patient sample was added to the confluent cell monolayers for 1 h at +37 °C, with gentle mixing. After that, MEM (supplemented with 2% foetal bovine serum, L-glutamine, and PenStrep) was added. The virus isolation trial cells were incubated at +37 °C, 5% CO_2_ and observed for cytopathic effects daily for 1 week followed by further passaging and cultivation for another 14 days.

### Sequencing

For INKV nested-RT-PCR, Invitrogen™ SuperScript™ III One-Step RT-PCR System with Platinum™ Taq DNA Polymerase (Thermo Fisher Scientific) with final concentration of 400 nM for primers: INKV-S-F1 (5′-GTAGTGTRCTCCACTTGAATACT-3′), INKV-S-R1 (5′-TGCTCCACTGAATACATTTAAC-3′) and INKV-S-F3 (5´-ATCATAAACCCRATTGCAGAATC-3′), INKV-S-R3 (5’-TTCTTGCAGCATCYCTAAGTC-3′), Lambert Cal/Bwa FW (5’-GCAAATGGATTTGATCCTGATGCAG-3′) and Lambert Cal/Bwa Rev. (5’-TTGTTCCTGTTTGCTGGAAAATGAT-3′) (F1 + R1, F1 + R3, F3 + R1, and Lambert FW + Lambert Rev. [16]) were used, with the nucleic acid template of 8 μl. Reaction mixes were subjected to RT-PCR amplification using the following cycling conditions: one cycle of 55 °C for 30 min and 94 °C for 2 min, followed by 40 cycles of 94 °C for 15 s, 55 °C for 30 s, and 68 °C for 1 min. Reactions were terminated with a final extension step at 68 °C for 5 min.

Protocol was continued with another PCR step using Thermo Scientific DreamTaq PCR Master Mix (2X) System with Thermo Scientific DreamTaq DNA Polymerase (Thermo Fisher Scientific) with a final concentration of 500 nM for primers: INKV-S-F2 (5′-CTATARAAGGCATACTTGGTTG-3′), INKV-S-R2 (5′-ATTATGCTGCTATGTTTATGGTC-3′), INKV-S-F4 (5′-GGAACAGAAATGTTYCTAGAAGT-3′) and INKV-S-R4 (5′-GCCYTAYCGTTGCCTAAGTG-3′) (F2 + R2, F2 + R4 and F4 + R2) were used, with One Step PCR product of 5 μl. Reaction mixes were subjected to PCR amplification using the following cycling conditions: one cycle of 95 °C for 2 min, followed by 40 cycles of 95 °C for 30 s, 55 °C for 1 min, and 72 °C for 2 min. Reactions were terminated with a final extension step at 72 °C for 10 min.

Products of RT-PCR amplification and regular PCR amplification were evaluated by electrophoresis in a 2.0% agarose gel in 40 mM Tris-acetate-1 mM EDTA buffer. Seven microliters of product were analysed for each amplified sample.

## Results

### Serology

Out of 1470 samples, 325 (22.1%) samples were anti-CSG orthobunyavirus IgG positive by IFA. In total, 15 acute cases with anti-INKV IgG and IgM antibodies were diagnosed using IFA: five in 2021, two in 2023, and eight in 2024. All these had a granular fluorescence pattern typical of early antibody responses against the N protein [12] in anti-INKV IgG and IgM IFA, indicating acute or recent infection. Among these 15 cases, two seroconversions were detected, one in the year 2021 and the other in 2023. Overall, the seroprevalence was 18.4% (108/588), 20.1% (98/488), and 30.2% (119/394) in 2021, 2023, and 2024, respectively.

MNT was carried out for all acute cases detected using IFA (anti-INKV IgG and IgM positives; *N* = 14), and for a panel of randomly selected sera representing old immunity (anti-INKV IgG positive but anti-INKV IgM negative; *N* = 30), and one serum negative in IFA. In IFA, the IgG antibodies create a diffuse fluorescence pattern typical of stronger responses to Gc protein than N, while in acute or recent infection, antibodies are against N protein only, and the fluorescence pattern is granular one [12, 13, 14]. All acute cases and patients with old immunity had neutralizing antibodies against INKV/CHATV, and negative serum was negative by MNT (Table 1). The titres ranged from ten to over 1280 for INKV and ten to 160 for CHATV. In one acute infection, there were similar titres for both INKV and CHATV; otherwise in all other samples, there were four-fold titre differences, indicating an INKV infection.

**Table 1.** Results of selected samples, i.e., all acute infections, of INKV and CHATV screening: MNT, MPLEX-RT-qPCR, and serology

Acute/recent infection (A); old immunity (O) or negative (N)		INKV-CHATV RT-qPCR	Microneutralization titre		Anti-INKV IFA	
ID (Year_Indiv._Sample order)		Pos/neg	INKV	CHATV	IgG	IgM
1_A	21_1_I^a^	Neg	10	<10	<20	Borderline +/−20, G+
	21_1_II^a^	Neg	80	<10	>20, G+	>20, G+
2_A	21_2_I	Neg	320	<10	>20, G+	>20, G+
3_A	21_3_I^a^	Neg	ND	ND	>20, G+	>20, G+
	21_3_II^a^	Neg	160	<10	>20, G+	>20, G+
4_A	21_4_I	Neg	80	10	>20, G+	>20, G+
5_A	21_5_I^a^	Pos	160	<10	20, G+	>20, G+
	21_5_II^a^	Neg	160	<10	>20, G/D+	>20, G+
6_A	21_6_I	Pos	ND	ND	<20	<20
7_A	23_1_I	Neg	80	80	>20, G+	>20, G+
8_A	23_2_I	Neg	ND	ND	<20	<20
	23_2_II	Neg	320	<10	>20, G+	>20, G+
9_A	24_1_I	Neg	40	<10	>20, G+	>20, G+
10_A	24_2_I	Neg	160	<10	>20, G+	>20, G+
11_A	24_3_I	Neg	320	<10	>20, G+	>20, G+
12_A	24_4_I	Neg	160	<10	>20, G/D+	>20, G+
13_A	24_5_I	Neg	160	10	>20, G+	>20, G+
14_A	24_6_I	Neg	160	<10	>20, G+	>20, G+
15_A	24_7_I	Neg	320	<10	>20, G/D+	>20, G+
16_A	24_8_I	Neg	320	10	>20, G+	>20, G+
1_O	21_6	Neg	1280	40	>20, D+	<20
2_O	21_7	Neg	320	20	>20, D+	<20
3_O	21_8	Neg	160	20	>20, D+	<20
4_O	21_9	Neg	320	20	>20, D+	<20
5_O	21_10	Neg	320	40	>20, D+	<20
6_O	21_11	Neg	20	<10	>20, D+	<20
7_O	21_12	Neg	80	10	>20, D+	<20
8_O	21_13	Neg	320	20	>20, D+	<20
9_O	21_14	Neg	160	20	>20, D+	<20
10_O	21_15	Neg	160	20	>20, D+	<20
11_O	21_16	Neg	80	10	>20, D+	<20
12_O	21_17	Neg	80	10	>20, D+	<20
13_O	21_18	Neg	640	80	>20, D+	<20
14_O	21_19	Neg	80	20	>20, D+	<20
15_O	21_20	Neg	160	20	>20, D+	<20
16_O	21_21	Neg	80	10	>20, D+	<20
17_O	21_22	Neg	160	20	>20, D+	<20
18_O	21_23	Neg	160	10	>20, D+	<20
19_O	21_24	Neg	>1280	40	>20, D+	<20
20_O	21_25	Neg	160	10	>20, D+	<20
21_O	21_26	Neg	160	10	>20, D+	<20
22_O	21_27	Neg	640	160	>20, D+	<20
23_O	21_28	Neg	160	20	>20, D+	<20
24_O	21_29	Neg	160	10	>20, D+	<20
25_O	21_30	Neg	160	20	>20, D+	<20
26_O	21_31	Neg	160	10	>20, D+	<20
27_O	21_32	Neg	160	10	>20, D+	<20
28_O	21_33	Neg	160	20	>20, D+	<20
29_O	21_34	Neg	160	10	>20, D+	<20
30_O	21_35	Neg	320	20	>20, D+	<20
1_N	21_37	Neg	<10	<10	<20	<20

<20, neg; >20, pos; 20, borderline; CHATV, Chatanga virus; D+, diffuse; G+, granular; G/D+, granular and some diffuse; INKV, Inkoo virus; MNT, microneutralization test; neg, negative; pos, positive.

a Paired samples from the same individuals (I) and (II).

### INKV-CHATV MPLEX RT-qPCR

To validate the specificity of MPLEX-RT-qPCR, 16 viral RNAs from other viruses, 20 EDTA blood, and 20 serum samples were analysed (Table 2). Those were all negative for INKV and CHATV (56/56, 100%). The limit of detections was 8.5 copies per PCR reaction (95% CI; Probit, SPSS) for INKV (strain KN3641) and 6.1 copies per PCR reaction for CHATV (strain Möhkö) (95% CI, Probit, SPSS). At a copy level of 254 with ten parallel reactions, the intra-assay repeatability was an average of 31.7 Ct, standard deviation (STDEV) of 0.4, and coefficient of variation (CV) of 1% for INKV (strain KN3641), and 571 copies, 29.7 Ct, STDEV of 0.4 and 1% (CV%) for CHATV (strain Möhkö). Ct values below 38 were considered positive.

**Table 2.** The non-INKV and CHATV viral RNAs (*N* = 16) used for INKV-CHATV MPLEX RT-qPCR validation

Virus	Strain	Source
Sindbis virus	Ockelby	In-house, HU
Tahyna virus	–	In-house, HU
Batai virus	–	In-house, HU
Yellow fever virus	17D vaccine strain	In-house
Yellow fever virus	Asibi	Robert Koch Institute^b^
West Nile virus	Eg101, lineage 1, clade A	In-house
West Nile virus	97–103	In-house
West Nile virus	B956, lineage 2	Robert Koch Institute^a^
Rift Valley fever virus	–	Robert Koch Institute^b^
Chikungunya virus	Caribbean	European Virus Archive
Chikungunya virus	R80422	Zeptometrix
Zika virus	MR766	In-house
Dengue virus type 1	Hawaii	Zeptometrix
Dengue virus type 2	New Guinea C	Zeptometrix
Dengue virus type 3	H87	Zeptometrix
Dengue virus type 4	H241	Zeptometrix

CHATV, Chatanga virus; HU, University of Helsinki; INKV, Inkoo virus.

a WNV B956; RNAs kindly provided by Arbovirus and Imported Viral Diseases Laboratory, CNM, ISCIII, Institut de Salud Carlos III, Centro Nacional de Microbiologia).

b RNA; Robert Koch Institute, kindly provided.

### Screening of serum samples with MPLEX-RT-qPCR, virus isolation attempts, and sequencing

Altogether 1417 out of 1470 serum samples were available for MPLEX-RT-qPCR as not all had enough sample left for extraction. Among MPLEX-RT-qPCR screened sera, two samples were repeatably INKV RNA positive by MPLEX-RT-qPCR. Both samples originated from the year 2021: one from September (MPLEX-RT-PCR Ct value 36.9) and other from October (Ct 35.7). The case from September had no anti-INKV antibodies, and no further samples were available from this case. The other one from October was also anti-INKV IgG and IgM antibody positive, indicating acute infection of INKV. No CHATV RNA-positive samples were found.

Virus isolation trials were attempted, but no viable virus could be cultivated. No CPE was observed, and all confirmatory RT-qPCR tests from supernatant were negative.

Due to the low viral RNA load detected by MPLEX-RT-qPCR and RNA degradation during storage and handling, including a total of three freeze–thaw cycles of extracted RNA, the nested S-segment amplifications were all negative. After being used in serology, MNT, virus cultivation, and MPLEX-RT-qPCR, the original samples were exhausted, and no further studies were carried out.

## Discussion

Mosquito-borne orthobunyaviruses INKV and CHATV are endemic in Finland [5, 9, 12]. As is the case with many other mosquito-borne viral infections, the symptoms are mostly mild and non-specific, and specific laboratory tests are needed in their diagnostics. Over 50 years ago, Brummer-Korvenkontio [4] reported that the seroprevalence of CSG viruses, such as INKV and CHATV, was increasing in the direction of northern Finland, starting from 16% in the south up to 69% in most northern parts of Lapland in the population studied. Putkuri et al. [12] showed that overall seroprevalence was 51.7% (*N* = 1287; samples from 2001 to 2004) in Finland, somewhat varying between different districts. In the same study, they showed that the seroprevalence was the lowest at the age group of 0–9 years (18.8%), thereafter increasing rapidly to over 40% at the age group of 10–19 years. In our study, conducted 20 years later using the same serological IFAs, and samples mostly acquired from adults, we showed that the overall seroprevalence was between 18 and 30% depending on the year screened (2021–2024). A limitation of this study was that there were no demographic or patient data, for example, age, sex, hospital districts, or symptoms, available (according to research and ethical permits). Due to this limitation, no age or district comparisons were carried out. Only two laboratories in Finland carry out diagnosis of SINV infections, and therefore, we know that also other than samples from the southern parts of Finland were in this study, and overall seroprevalence can be compared at some level to the ones from 20 years ago.

In our study, acute infections caused by orthobunyaviruses were found annually, and neutralization assays indicated that all but one were caused by INKV. In Finland, INKV and CHATV infections are rarely diagnosed, although serological tests are available in diagnostic laboratories. During the years 2021–2024, there were no reports of laboratory-diagnosed INKV or CHATV virus infections in Finland (Finnish Institute for Health and Welfare, 2024). The lack of using specific diagnostic tests is likely due to a lack of knowledge of these endemic viruses, and the recognition of the infection is further hampered by non-specific symptoms.

In the early phase of the disease, the symptoms of INKV, CHATV, and SINV infections can be similar, although a rash has not been commonly reported with INKV and CHATV infections. In Putkuri et al. [9], INKV infections were more often detected in the younger age groups than CHATV infections, which were more often found in the adult population. In Europe, an INKV and CHATV-related Tahyna orthobunyavirus (TAHV) is endemic and causes similarly febrile disease in late summer and early autumn, with, e.g., fever, gastrointestinal disorders, and influenza-like or “summer flu”-like symptoms [17, 18]. Symptoms caused by INKV infection, the ones caused by TAHV, are milder in adults than in children [9, 18]. Most often, INKV, TAHV, and CHATV infections are subclinical or represent mild febrile illness, and only rarely complications are seen in immunocompetent persons; these involve CNS infections [9, 18].

Currently, diagnosis is carried out using only serological methods. In this study, a novel molecular tool was developed and validated to detect and discriminate the INKV and CHATV RNAs from each other in one reaction using a multiplex one-step RT-qPCR assay format with good sensitivity. Using this method, we detected INKV RNA in two patients, suggesting low viraemia. Both cases were from the year 2021, and one of the cases was also confirmed by serological assays, including MNT, to be an INKV infection. The level of viraemia detected was low, and no viruses were isolated from the original serum samples. However, these samples were frozen and thawed several times before isolation attempts, as they were not originally meant for virus isolation trials, and such repeated freeze–thaw cycles can affect the infectivity of the virus. Although the MPLEX-RT-qPCR sensitivity was validated to be good and two cases were detected, further studies are needed to evaluate the diagnostic value of this molecular assay together with the serodiagnosis, especially in severe disease forms like CNS infections and cerebrospinal fluid samples, as well as aspects of molecular epidemiology.

There were, altogether, six acute INKV infections in 2021 (total *N* = 588; individuals 572, 6/572, 1.1%), one INKV and one INKV/CHATV infection in 2023 (total *N* = 488, individuals 469; 2/469, 0.4%), and eight INKV infections in 2024 (total *N* = 394, individuals 377; 8/377, 2.1%). Months with the highest occurrence of CSG infections were August and September, independent of the year. The year 2024 might have been somewhat different since fewer samples were sent to the diagnostic laboratory, yet more acute INKV infections were detected and seroprevalence was higher than in previous years of 2021 and 2023. Most cases were detected during September until October, especially in 2024. Only one acute INKV infection was detected during the earlier summer, in June (year 2021).

Nikiforova et al. [19] screened 420 serum samples collected in year 2018 from patients with viral infection of unknown aetiology in Astrakhan, Russia, for different arboviruses, and 41 cases out of 420 were positive for arboviral nucleic acids. Out of those 41, 32 were initially misdiagnosed as having a respiratory infection, and half of them just presenting with fever, myalgia, and vomiting. They detected six SINV infections and 27 orthobunyavirus infections: two INKV, one Tahyna virus, and 24 Batai virus infections. In our study, we describe the first detection of INKV RNA from patient serum samples in Finland. Before this study, only serological methods were available for diagnosing INKV and CHATV infections in Finland. In our study, the demographic and clinical data from patients, however, were not available, and therefore, further studies would be needed for obtaining information on the symptom diversity, especially in mild disease forms of INKV and CHATV, and for comparing those with the symptoms of SINV.

At present, very little is known about the viraemia in acute INKV or CHATV disease, and no larger screening studies have been published or otherwise made available. Commonly, it is thought that viraemia levels remain too low in CSG infections in larger mammals to infect new mosquitoes, and these viruses are rarely tested from human samples, especially from immunocompetent patients, by molecular methods [20, 21, 22].

We conclude that orthobunyavirus infections should be suspected during summertime in patients with non-specific symptoms like fever, myalgia, and vomiting in Finland. If the patient is not having typical symptoms for SINV infection, like arthritis, then clinicians should also consider INKV or CHATV infections. In our study, seroprevalence has declined somewhat compared to previous years. This raises questions: are there changes in human behaviour or in enzoonotic cycles? Based on the observed seroprevalence and the few acute cases detected, the disease burden, and consequently the economic burden, remains low. However, this may change in the future, and these viral infections should not be overlooked in clinical settings. Nevertheless, in Finland, orthobunyaviruses remain neglected causes of febrile diseases during the mosquito season.

## Data Availability

All data are shown in manuscript, and more detailed data are available upon a request.
